# Cell and gene therapies: moving from research to clinic

**DOI:** 10.1186/1479-5876-8-31

**Published:** 2010-03-29

**Authors:** David F Stroncek, Raj K Puri

**Affiliations:** 1 Department of Transfusion Medicine, Clinical Center, NIH, Bethesda, Maryland, USA; 2 Center for Biologics Evaluation and Research, Food and Drug Administration, Bethesda, Maryland, USA

## Editorial

Cell and gene therapy clinical trials began more than 40 years ago. At some institutions cellular therapy laboratories were started to support marrow transplantation. These early laboratories removed red blood cells and plasma from aspirated bone marrow that was used for allogeneic transplants, cyropreserved autologous marrow, depleted T cells from allogeneic grafts and depleted leukemic or cancerous cells from autologous grafts [1]. At other institutions cellular therapy laboratories were started to isolate and expand tumor infiltrating lymphocytes (TIL) that were used as investigational treatments for patients with melanoma or to prepare transfected autologous lymphocytes to treat severe combined immune deficiencies.

For many years cellular and gene therapies were primarily highly experimental therapies which were developed and used in highly specialized academic health care centers. Now these forms of therapies are used in numerous clinical trials throughout the US and worldwide. While the field has advanced, progress has been slow. Some therapies have not been effective and some have been associated with unacceptable adverse out comes. However, both cell and gene therapies have now become important potential therapies for incurable diseases.

Hematopoietic stem cell transplants have changed dramatically and have become very successful for hematopoietic reconstitution. Hematopoietic stem cell transplants now make use of marrow, mobilized peripheral blood stem cells and umbilical cord blood for transplants involving HLA matched siblings and unrelated subjects as well as autologous transplants. Recently, there have been important advances in immune therapy of cancer. Immune therapy for melanoma protocols that involve TIL make use of lymphodepletion and autologous CD34+ cell rescue have been reported to result in a greater than 50% objective clinical response rates [2]. Gene therapy is being used as investigational treatment for severe combined immune deficiency (SCID), Leber's Congenital Amaurosis (LCA) and chronic granulomatous disease (CGD) [3] and may soon be used in clinical trials to treat sickle cell disease.

The successful clinical results of some cellular and gene therapy clinical trials and the increased understanding of immunology, cancer, and stem cell biology have lead to the development of many potential new therapies. Natural killer (NK) cells and dendritic cells (DCs) are important parts of many cancer immune therapy investigational protocols. Genetically engineered T cells and DCs are being tested for immune therapy for cancer. Vectors containing tumor reactive T cell receptors are being introduced into T cells. Chimeric receptors containing antibodies specific to antigens expressed by leukemic cells along with T cell costimulatory molecules are being transferred into T cells that are being used therapeutically. Artificial antigen presenting cells are being made by introducing costimulatory molecules into cell lines and these cells are being used to expand cytotoxic T cells *in vitro*.

Regenerative medicine is an emerging new field. Marrow and mobilized PBSCs injected into ischemic myocardium was been reported to increase cardiac function [4]. Meschenchymal stem cells or bone marrow stromal cells (BMSCs) are also being used to as investigational treatments for ischemic heart disease. BMSCs are also being tested for the treatment of acute renal failure, nerve injury, acute GVHD and autoimmune disease [5]. Induced pluripotent stem (IPS) cells harbor great potential for regenerative medicine applications and for a number of hematopoietic and immune disorders. Work with IPS cells is moving quickly, but the routine clinical application of IPS cells is still many years away.

Translational studies have been and will continue to be critical to progress in cellular and gene therapy. The converging nature of gene therapy, immune therapy for cancer, HSC transplantation, regenerative medicine and tissue engineering make the rapid and widespread exchange of information essential. The goal the JTM Cell and Gene Therapy Section is to advance this field by reporting the results of translational medicine studies and by being a forum for the exchange and discussion of new information, ideas and hypothesis. We welcome contributions from all those participating in this field; clinicians, scientists, and engineers from academia, industry and the regulatory community.
